# Primary Prevention of Cannabis Use: A Systematic Review of Randomized Controlled Trials

**DOI:** 10.1371/journal.pone.0053187

**Published:** 2013-01-11

**Authors:** Melissa M. Norberg, Sarah Kezelman, Nicholas Lim-Howe

**Affiliations:** National Cannabis Prevention and Information Centre, University of New South Wales, Randwick, New South Wales, Australia; Maastricht University Medical Centre, The Netherlands

## Abstract

A systematic review of primary prevention was conducted for cannabis use outcomes in youth and young adults. The aim of the review was to develop a comprehensive understanding of prevention programming by assessing universal, targeted, uni-modal, and multi-modal approaches as well as individual program characteristics. Twenty-eight articles, representing 25 unique studies, identified from eight electronic databases (EMBASE, MEDLINE, CINAHL, ERIC, PsycINFO, DRUG, EBM Reviews, and Project CORK), were eligible for inclusion. Results indicated that primary prevention programs can be effective in reducing cannabis use in youth populations, with statistically significant effect sizes ranging from trivial (0.07) to extremely large (5.26), with the majority of significant effect sizes being trivial to small. Given that the preponderance of significant effect sizes were trivial to small and that percentages of statistically significant and non-statistically significant findings were often equivalent across program type and individual components, the effectiveness of primary prevention for cannabis use should be interpreted with caution. Universal multi-modal programs appeared to outperform other program types (i.e, universal uni-modal, targeted multi-modal, targeted unimodal). Specifically, universal multi-modal programs that targeted early adolescents (10–13 year olds), utilised non-teacher or multiple facilitators, were short in duration (10 sessions or less), and implemented boosters sessions were associated with large median effect sizes. While there were studies in these areas that contradicted these results, the results highlight the importance of assessing the interdependent relationship of program components and program types. Finally, results indicated that the overall quality of included studies was poor, with an average quality rating of 4.64 out of 9. Thus, further quality research and reporting and the development of new innovative programs are required.

## Introduction

Cannabis is the most widely used illicit drug worldwide [1] and its use is particularly prominent among adolescents and young adults [2], [3]. For example, in 2010, 15.7% of Australian youth aged 14 to 19 years had used cannabis in the last 12 months, whereas only 4.7% of Australians over 40 years of age had used [3]. Early initiation of cannabis use is associated with more intensive cannabis use [1], [4], and thus, a greater likelihood of developing dependence [5]. In addition, early initiation is associated with an increase in other health risk behaviors [6], [7], poor educational outcomes [8], [9], impaired cognitive functioning [10], and an increased risk of mental health issues [11], [12]. The risks associated with cannabis use are becoming increasingly concerning given recent increases in cannabis use among young people (12.9% of 14 to 19 year olds had used cannabis in 2007) [13]. Furthermore, only 29% of individuals who meet criteria for cannabis dependence seek treatment [14]. Of those who seek treatment, only 31 to 36% experience clinically significant reductions in their use [15], [16]. Given the extent of associated adverse effects for early and heavy use and the less than optimal treatment outcomes, primary prevention of cannabis use is critical.

As few researchers have posited what program components may lead to effective prevention of cannabis use, examination of the broader substance use literature may be informative in identifying key components for consideration. When attempting to prevent substance use in general, researchers have highlighted that theoretical models, program design, program facilitators, the developmental stage of participants, and program duration are key variables to consider for program efficacy [17]. Programs that have adopted psychosocial skills-based approaches with interactive designs have performed better than affective or knowledge-based approaches utilizing non-interactive, didactic designs [18]–[20]. Furthermore, programs that have adopted non-teachers as facilitators consistently have outperformed programs utilizing teachers [17], [21]. Family interventions as non-school-based interventions [22]. While evidence for optimal duration and timing of program delivery is largely inconclusive [17], [20], evidence from a systematic review on substance use prevention indicates that the implementation of booster sessions leads to greater and longer lasting program efficacy [23]. While these substance use literature findings are informative, further research is needed to confirm that these patterns of efficacy are upheld when cannabis use outcomes are assessed independently.

To date, two meta-analyses and one systematic review have assessed cannabis use prevention specifically [24]–[26]. All three reviews concluded that prevention strategies had potential for effectively reducing cannabis use in adolescents. In accordance with the broader substance use literature, interactive program designs were associated with greater program efficacy. Similarly, programs utilizing multiple theoretical models to inform program design and those adopting non-teacher facilitators (i.e., peer leaders or mental health counselors) were found to be more efficacious in reducing cannabis use. Conclusive statements regarding other key program characteristics (e.g., program duration, program size, and specifics of program theory) were not tenable as inconsistent evidence was presented across studies and/or limited data was available to make meaningful analyses [26].

These reviews also are limited in that they focused exclusively on school-based (uni-modal) programs and did not differentiate between universal and targeted intervention programming. Recent developments in the primary prevention literature implicate the importance of multi-modal approaches that utilize family, peer, community, and school-based components [27]. A review that considers multi-modal and uni-modal approaches will allow for a more rigorous assessment of substance use prevention programs. Furthermore, research is needed to determine the relative efficacy of universal (i.e., programs that attend to the general student population) versus targeted/selective programs (i.e., those that attend to high-risk populations). Some authors suggest that prevention strategies are unlikely to be universally effective because adolescents are not universally at risk [28]. Conversely, other authors have demonstrated that universal programs are effective for a variety of youth, including those at high-risk for substance use [29]. Thus, a consideration of various types of approaches and populations will enable a more rigorous review of prevention components and the extent of their efficacy.

There are a number of methodological considerations regarding the aforementioned meta-analyses and systematic review. All three reviews assessed quasi-experimental as well as experimental study designs. While this approach is reasonable, a sole focus on randomized controlled trials (RCTs) is suitable for systematic review as RCTs are more likely to provide unbiased information [30]. In addition, meta-analytic procedures are inappropriate if the heterogeneity is too large [31]. Marked clinical, methodological, and statistical heterogeneity was evident across studies included in all three reviews. Adopting meta-analytic techniques in these instances may be misleading, therefore a systematic review which offers a qualitative approach may be more appropriate [30]. In addition, despite the marked heterogeneity of effect sizes, only the systematic review [26] included an assessment of study quality. This review, however, included only three programs with cannabis use outcomes due to restricted inclusion criteria, which limited their capacity to assess outcomes and limited the generalizability of their results. A review that considers study quality while maintaining a rigorous inclusion criteria pertaining to study design may enable a more comprehensive understanding of program efficacy.

As such, the current study sought to extend existing literature by conducting a systematic review of cannabis use treatment outcomes in primary prevention research. The current review sought to determine the relative effectiveness of universal, targeted, uni-modal, and multi-modal programs, and to explore whether the effectiveness of these programs in deterring cannabis use differed as a function of participant age, program facilitators, program duration, program booster sessions, and program content. This study also assessed if study quality was associated with program effectiveness. The expectation was that a more comprehensive understanding would facilitate a more strategic and informed prevention approach to address the increasingly problematic prevalence of cannabis use in adolescent and youth populations.

## Methods

### Search procedures and selection criteria

We conducted a comprehensive search of eight electronic databases (EMBASE, MEDLINE, CINAHL, ERIC, PsycINFO, DRUG, EBM Reviews, and Project CORK) to identify relevant studies published between 1987 and January 2011. Key search terms were “cannabis* OR marijuana* or tetrahydrocannabinol” AND “adolescen* OR child* OR youth* OR young adult* OR student*” AND “prevention* OR early intervention* OR program evaluation* OR school-based*” (for an example of a full electronic search strategy see Appendix S1). This search identified 1975 records, of which 284 were duplicates. An additional 33 articles were discovered using the reference lists of articles identified through the search. Of the 1724 available records, an independent review of the titles and abstracts performed by two authors demonstrated that 1617 did not assess cannabis use or primary prevention and were omitted. Thus, 107 full-text articles were assessed for eligibility. Eligibility was assessed independently by two authors and discrepancies were resolved by a third author.

**Figure 1:**
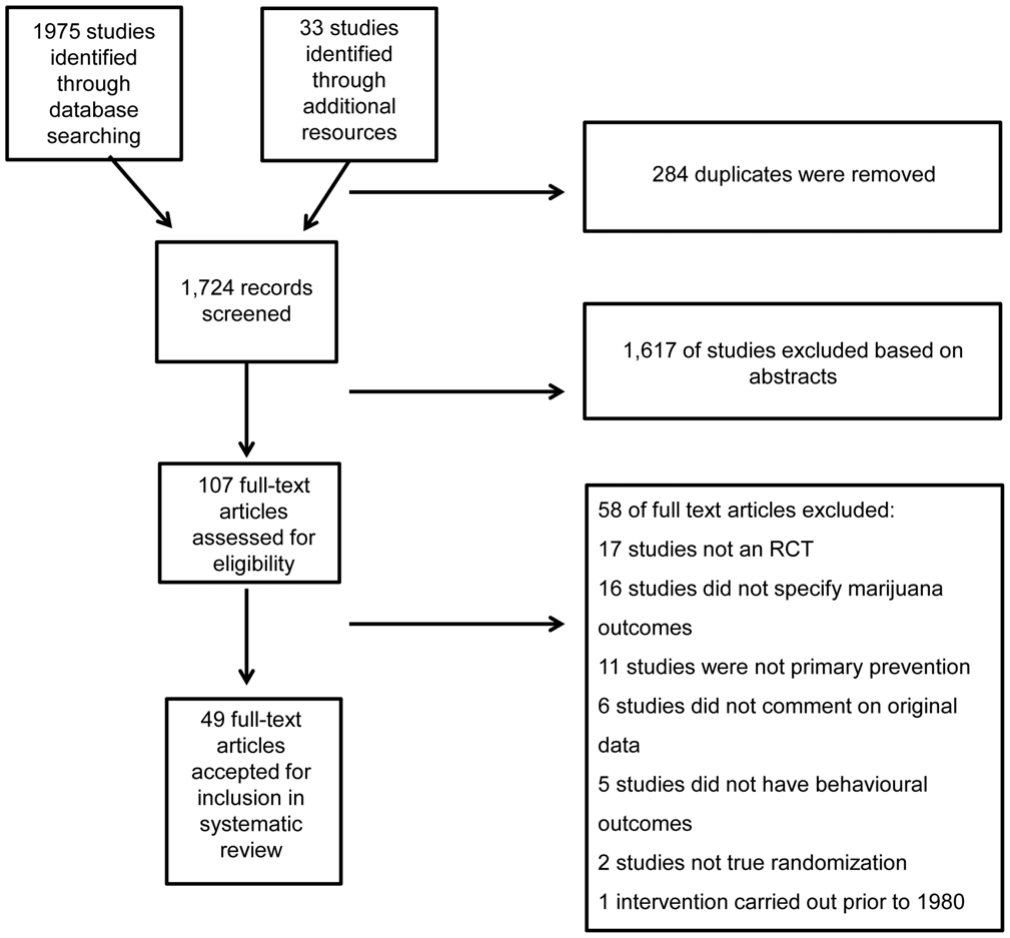
Flow-diagram presenting the process of identification for study eligibility.

To be eligible for inclusion, studies must have (a) assessed cannabis use, (b) implemented the program as a primary prevention effort, (c) examined young people (children, adolescents, or young adults [≤24 years]), and (d) presented original data. In addition, studies had to be RCTs published in a peer-reviewed journal in English. Overall, 49 RCTs reached the standards stipulated by the inclusion criteria. Figure 1 presents a flow-diagram of the selection process.

### Data extraction

Two authors independently extracted data from eligible studies and discrepancies were resolved by discussion with a third author. Data information collected included the study design (control group, comparison groups), demographic characteristics (age, gender, and ethnic distributions), intervention components, theoretical foundations, details of the outcome measures, statistical analysis procedures, and cannabis-specific outcomes. When a study did not report participants' ages, age was approximated from available information. For example, when a study reported a seventh grade sample, the average age of seventh grade students, 12 years, was used as an approximation. Additionally, when an age range was presented, the middle value of that range was documented.

### Quality assessment

Study quality was assessed using an adapted version of the EPOC Risk of Bias criteria [32]. As this review only included RCTs two EPOC criteria (allocation sequence and allocation adequacy) were deemed inappropriate. Three additional criteria (adherence adequacy, exposure adequacy, and reliability of outcome measures) were identified as important criteria for primary prevention and were added. These amendments resulted in a total of nine quality criteria that assessed study methodology, fidelity of program implementation, appropriateness of outcome measures, and statistical procedures (for full details see Appendix S2). Quality criteria were scored dichotomously, where 0 = unclear/did not fulfill criteria and 1 = did fulfill criteria. Thus, quality ratings could range from 0 to 9, with higher scores indicating better quality. As with study eligibility, quality was independently assessed by two authors and all differences were resolved by discussions with a third author.

### Data Synthesis

To adequately compare the effectiveness of the primary prevention programs, standardized effect size estimates were calculated using Cohen's *d* [33]. Cohen's *d* is defined as the difference between two means divided by the pooled standard deviation. According to [33], an effect size can be described as being small, moderate, or large, corresponding approximately to a 20%, 50%, and 80% change respectively. All effect size values were presented in the desired direction, such that positive values reflect a better outcome for treatment participants compared to controls. In addition, 95% confidence intervals (CIs) for each effect size value were calculated. Effect size and CI values not only facilitate a conventional statistical significance test, but also provide detailed information on effect magnitude and test precision, enabling an assessment of clinical significance [34]. For this review, a 95% CI that did not include zero was considered statistically significant. It is important to note that statistical significance was often inconsistent within studies for different outcome measures at different time points. Thus, a single study could be assessed as having both significant and non-significant findings. Furthermore, multiple effect size statistics (e.g., different outcome measures, different follow-up periods) were often included for a single study, as information (e.g., degrees of freedom and inter-correlation values) needed to combine these effects was often not available [35].

The studies included in this systematic review widely varied in their statistical reporting. As a result, multiple techniques were applied to compute Cohen's *d* and 95% CIs. When available, Cohen's *d* and CIs were reported as stipulated in the published papers or were calculated from means and standard deviations provided in the published papers. On occasion, however, the reported effect size estimate did not have corresponding confidence limits, or means and standard deviations were not reported. In these cases, an online effect size calculator [36] or appropriate conversion formulae were applied to estimate Cohen's *d* from standard errors and mean values, odds ratios, *F*-values, or chi-square values. For example, a ln(odds ratios) can be converted to an effect size estimate by dividing by 1.81 [37]. When group sample size values were unknown group equivalency was assumed. When these conversion formulae were not applicable, authors were contacted to provide means, standard deviations, and sample size values to allow for calculation of Cohen's *d* and CIs. If authors were unable to provide required information or failed to reply to two email communications their study articles were subsequently excluded from this systematic review (*n* = 21, for a list of excluded studies see Appendix S3).

A formal meta-analysis was not conducted as marked heterogeneity of study design, participant characteristics, intervention components, outcome measures, effect statistics, and study quality rendered a meta-analysis meaningless [30]. As a result, a narrative synthesis approach was applied. Included studies were categorized on a number of program components in order to effectively assess the relative efficacy of universal, targeted, uni-modal, and multi-modal program designs. These syntheses considered statistically significant and non-significant results separately, as well as differential median effect size magnitudes for statistically significant outcomes.

## Results

Overall, 28 articles were included in this systematic review, representing 25 unique RCTs. Most of the included studies were conducted in the United States (*n* = 21), with the remaining studies implemented in Australia (*n* = 2), the United Kingdom (*n* = 1), and Europe (*n* = 1). The number of participants varied considerably across prevention programs with baseline sample size ranging from 64 to 7079 (*M* = 1933; *SD* = 2068). Although we attempted to extract data pertaining to theoretical foundations, a meaningful analysis was not feasible as insufficient information was reported to adequately differentiate programs based on theory. Tables 1, 2, 3, 4 present the program characteristics, effectiveness data, and study quality ratings for the universal and targeted prevention programs subdivided by uni-modal and multi-modal program design.

**Table 1:** Program characteristics for universal uni-modal programs.

Citation	*N*	Sample (age)	Program	Comparison Group	Control group	Program Leader	No. Sessions	Outcome Measures	Post-test effect-size (months)	Follow-up effect-size (months)	Quality Rating
Botvin et al. [55]; Botvin et al. [54]	1311	7^th^ grade (NA)	LST	LST Peer, LST Teacher, LST Peer+Booster, LST Teacher+Booster	NT	PL, T	20, 20, 20+10 boosters, 20+10 boosters	Monthly use, Weekly use, Use index^h^	0.13* c d, 0.13* c d	0.1^c^ ^d^(12), 0.11^c^ ^d^(12)	3
Botvin et al. [72]; Botvin et al. [73]	5954	7^th^ grade (NA)	LST^a^	LST (E1), LST (E2)	TU	T	15+10 boosters (yr1)+5 boosters (yr2)	Frequency of use (36), Monthly use (72), Weekly use (72)	NA	0.11* d(36), 0.01^f^(72), 0.09* f(72), 0.09* d(36), 0.01^f^(72), 0.05^f^(72)	4
Dent et al. [74]	1208	High School (14–17)	TND	-	TU	HE	9	Frequency of use past month	NA	0.09^d^ (12)	4
Newton et al. [41]	764	High School (*M* = 13.08)	CSM	-	TU	T	12	Frequency of use past 3 months	−0.17	0.16(6), 0.23*(12)	6
Ringwalt et al. [75]	6090	6^th^ & 7^th^ grade	ALERT Revised	-	DI	T	11+3 boosters	Lifetime use, 30-day use	0.04^e^, 0.03^e^	NA	5
Rohrbach et al. [76]	3346	Regular & Continuation High School (*M* = 14.1)	TND^b^	IMP-Support, Regular	TU	T	12	30-day use	NA	0.14* c e (12)	4
Werch et al. [39]	604	High School (*M* = 15.24)	SPORT	-	MC	HP	1	30-day use, Stages of initiation	0.14^f^ (3), 0.11^f^ (3)	0.11^f^ (12), 0.17^f^ (12)	6

*Note*. LST = Life Skills Training, TND = Towards No Drug Abuse, CSM = Climate Schools Model. NT = No Treatment, TU = Treatment as usual, DI = Delayed Intervention, MC = Minimal Contact Control. PL = Peer Leader, T = Teacher, HE = Health Educator, HP = Health Professional. NA = Not applicable/Not available.

a This intervention assessed differential training for teachers: the E1 condition utilised a 1-day teacher workshop with implementation feedback, and the E2 condition utilised a videotape providing no feedback.

b This intervention assessed differential training for teachers: the IMP-support refers to comprehensive implementation support, and the REGULAR refers to regular workshop training only.

c Results were collapsed across comparison groups and analyses were treated as all intervention versus control.

d Cohen's d calculated using F value.

e Cohen's d calculated using odds ratio.

f Cohen's d calculated by converting standard error to standard deviation.

* Statistically significant as 95% CI does not contain zero.

**Table 2:** Program characteristics for universal multi-modal programs.

Citation	*N*	Sample (age)	Program	Comparison Group	Control group	Program Leader	No. Sessions	Outcome Measures	Post-test effect-size (months)	Follow-up effect-size (months)	Quality Rating
Bond et al. [77]	2678	Secondary School (*M* = 14)	GP	-	U	T	20 (median)	Any use past 6 months	0.01^b^	−0.03^b^(12), 0.12^b^(24)	3
Ellickson et al. [78]	4689	7^th^ & 8^th^ grade (NA)	ALERT Revised	ALERT Revised	TU	T	11+3 boosters	Lifetime use, Monthly use, Weekly use	NA	0.15* (18), 0.08* (18), 0.08* (18)	5
Faggiano et al. [52],[53]	7079	Junior High School (12–14)	EU-Dap	Basic, Basic+Peer, Basic+Parent	NT	T, T+PL, T+P	12–15	Any use, Frequent use	0.14* b c (3), 0.15* b c (3)	0.10^b^ ^c^ (18), 0.17* b c (18)	5
Johnson et al. [79]	1607	6^th^ & 7^th^ Grade (NA)	MPP	-	MC	T	10	30 day use	NA	0.08^e^ (36)	4
Schinke et al. [40]	514	Youth (10–12, *M* = 11.5)	-	CD-Rom, CD-Rom+ P	U	NA^a^, P	10+annual booster	Past 30 day use	1.96*, 2.61*	2.86* (12), 1.63* (24), 1.99* (36), 5.26* (12), 1.98* (24), 2.38* (36)	8
Spoth et al. [44]	667	6^th^ Grade (NA)	ISFP & PDFY	ISFP, PDFY	MC	PW	5, 7	New user proportion	NA	0.90* (48), 0.49* (48)	4
Spoth et al. [80]	1664	7^th^ grade (NA)	LST & SFP 10–14	LST Only, LST+SFP 10–14	TU	T, T & PW	LST:15+5 booster, SFP: 7+4 booster	New user proportion-	NA	0.68* (12), 0.75* (12)	6
Werch et al. [51]	448	8^th^ grade (*M* = 13.4)	STARS & STARS Plus^l^	STARS, STARS Plus	MC	HP	1+8 Postcards, Postcards Only	30-day use	0.06^d^(3), −0.03^d^(3)	NA	4

*Note*. GP = Gatehouse Project, EU-DAP = European Drug Abuse Prevention, MPP: Midwestern Prevention Program, ISFP = Iowa Strengthening Families Program, PDFY = Preparing for the Drug Free Years, LST = Life Skills Training, SFP 10–14 = Strengthening Families Program: For Parents and Youth 10–14. U = Unclear/Unspecified, TU = Treatment as usual, NT = No Treatment, MC = Minimal Contact Control. T = Teacher, PL = Peer Leader, P = Parent, NA = Not applicable/Not available, PW = Project Worker, HP = Health Professional.

a This is a CD-Rom based intervention program facilitator is thus not applicable.

b Cohen's d calculated using odds ratio.

c Results were collapsed across comparison groups and analyses were treated as all intervention versus control.

d Cohen's d was reported by study authors.

e Cohen's d calculated using chi-square.

* Statistically significant as 95% CI does not contain zero.

**Table 3:** Program characteristics for targeted uni-modal programs.

Citation	*N*	Sample (age)	Program	Comparison Groups	Control Group	Program Leader	No. Sessions	Outcome measures	Post-test effect size (months)	Follow-up effect-size (months)	Quality Rating
Conrod et al. [81]	732	High-risk personalities^a^ (*Mdn* = 14)	-	-	TU	HP	2	Frequency of use past 6 months	NA	−0.05^h^ (6), 0.06^h^ (12), 0.2* h (18), 0.2* h (24)	4
Elliot et al. [46]	928	Female High School Athletes (*M* = 15.35)	ATHENA	-	MC	PL	8	Last year use, Lifetime use	NA	0.74* h (12–36), 0.53* h (12–36)	6
Naar-King et al. [42]	64	HIV Positive Youth (16–25)	HC:MET	-	DI	HP	4	TLFB	0.34^f^ (3)	NA	5
Palinkas et al. [50]	296	High-risk female^b^ (14–19, *M* = 16)	PALS	FOL, FOL+PALS	NA^d^	HP	16, 32	Frequency of use past 3 months	NA	0.19^h^ (3)	4
Schinke et al. [47]	916	Adolescent females and their mothers (*M* = 12.76)	-	-	NT	P	9+1 booster	Frequency of use past month	NA	0.14* (12), 0.20* (24)	8
Schwinn et al. [49]	236	Adolescent females (*M* = 14)	RT	-	NT	NA^e^	12	Frequency of use past month, frequency of use past week	NA	0.35* i (6)	7
Stanton et al. [48]	817	African American youth (13–16)	ImPACT^c^	FOK, FOK+ImPACT, FOK+ImPACT+Boosters	-	PW	8, 8, 12	Frequency of use in past 6 months	0.10^g^ (6)	0.12^g^ (12), −0.04^g^ (18), 0.20* g (24),	5

*Note*. HC: MET = Healthy Choices: Motivational Enhancement Therapy, PALS = Positive Adolescent Life Skills, RT = Real Teen, ImPACT = Informed Parents and Children Together, FOL = Facts of Life, FOK = Focus on Kids. TU = Treatment as usual, MC = Minimal Contact Control, DI = Delayed Intervention, NA = Not applicable/Not available, NT = No Treatment. HP = Health Professional, PL = Peer Leader, P = Parent, PW = Project Worker. Other: TLFB = Timeline Follow-back.

a Participants defined as high-risk personalities if they scored 1 standard deviation higher than the school mean on 1 of 4 subscales of the Substance Use Risk Profit Scale: hopelessness, anxiety sensitivity, impulsivity, or sensation seeking.

b High-risk females were defined as pregnant adolescents who were using drugs OR who were at risk for using drugs, and for non-pregnant adolescents who were either using drugs OR at risk for using drugs AND who were at risk for pregnancy, where risk was determined using the Problem Oriented Survey Instrument for Teenagers (POSIT).

c The authors did not employ a “true” control group for ethical reasons, i.e. not providing an adequate level of standard care to all participants. Thus, the study design compared the new treatment of interest (ImPACT) to an established treatment (FOK: Focus on Kids).

d The authors did not employ a “true” control group to minimize the possibility of a Hawthorn Effect. Thus, the study compared the new treatment (PALS) to an established normative education treatment (FOL: Fast of Life).

e This is a computer based prevention program, program facilitators are thus not applicable.

f Cohen's d reported by author.

g Results were collapsed across comparison groups and analyses were treated as all intervention versus control.

h Cohen's d calculated from odds ratio.

i Cohen's d calculated from F-value.

* Statistically significant as 95% CI does not contain zero.

**Table 4:** Program characteristics for targeted multi-modal programs.

Citation	*N*	Sample (age)	Program	Comparison Groups	Control Group	Program Leader	No. Sessions	Outcome measures	Post-test effect size (months)	Follow-up effect-size (months)	Quality Rating
Griffin et al. [82]	199	African American Middle school population (NA)	BRAVE	-	NT	CRM	18–27	Frequency of use past 30 days	0.30^b^^-^	NA	3
Grossbard et al. [38]	1275	1^st^ yr college who participated in high school athletics (NA)	BASICS	BASICS Only, Parent Only, Combined BASICS+Parent	DI	PL, Mailout , PL+Mailout	1, 0, 1	Frequency of use past 30 days	NA	−0.07 (10), 0.06 (10), 0.14 (10)	2
Hecht et al. [43]	4234	Mexican American students (11–18, M = 12.53)	Keepin' it R.E.A.L	Mexican American, Black/White, Multicultural^a^	TU	T	10+U boosters--	Quantity of use past 30 days, Frequency of use past 30 days	0.04 (2), 0.02 (2), −0.05(2), −0.06 (2), −0.07 (2), −0.07 (2)	0.09*, 0.05 (8), 0.12*, 0.11*(14), −0.03, −0.04 (8), 0.02, 0.00 (14), −0.04, −0.05 (8), 0.07*, 0.07 (14)	1

*Note.* Programs: BRAVE = Building Resiliency and Vocational Excellence, BASICS = Brief Alcohol Screening and Intervention for College Students, R.E.A.L = Refuse, Explain, Avoid, Leave. NT = No Treatment, DI = Delayed Intervention, TU = Treatment as usual. CRM = Community Role Model, PL = Peer Leader, T = teacher. NA = Not applicable/Not available.

a This project assessed 3 cultural versions of an intervention (1) Mexican American, (2) Black/White, and (3) a multicultural version which incorporated aspects of the first two.

b Cohen's d calculated from F-value.

* Statistically significant as 95% CI does not contain zero.

### Cannabis specific program content

The majority of studies (60%, *n* = 15) did not report cannabis-specific content components. Rather, many of the studies reported targeting substance use in general or tobacco, alcohol, and other drugs in combination as content areas. Three studies (11%; [38], [39], [40]) were alcohol specific programs and thus did not address cannabis use in their program content (but did measure cannabis use as an outcome). While six studies (24%) specified that cannabis-specific content was included in their program, only one study (4%; [41]) specified an entire module devoted to cannabis. Of those programs reporting cannabis specific program content three (50%) reported statistically significant findings (*d* = 0.09 to 0.22, *Mdn* = 0.12) in comparison to 12 (63%) studies that did not include cannabis specific content (*d* = 0.07 to 5.26, *Mdn* = 0.30).

### General program content

The vast majority (84%, *n* = 21) of the 25 included studies reported some form of psychoeducation. Other typical content included social skills training (64%, *n* = 16), risk resiliency/refusal skills training (60%, *n* = 15), and decision making skills training (40%, *n* = 10). Six studies (24%) covered all four content areas, of which five reported statistically significant results (*d* = 0.09 to 5.26, *Mdn* = 1.19). Five studies covered three of the four content areas, all of which reported statistically significant results (*d* = 0.07 to 0.90, *Mdn* = 0.16, *n* = 5). Nine studies included content for only two areas, of which four reported statistically significant results (*d* = 0.08 to 0.74, *Mdn* = 0.15), and four studies reported inclusion of only one area, of which one was significant (*d* = 0.20). One study [42] used motivational enhancement therapy and did not apply any of the aforementioned content areas, though results were not statistically significant. Further analysis of specific content components was not possible.

### Outcome measures and assessment intervals

The vast majority (92%; *n* = 23) of the 25 included studies reported outcome measures relating to frequency of use. These measures were dichotomous or continuous and time intervals measured ranged from weekly use to lifetime use, with some studies assessing multiple time periods (e.g., monthly and lifetime use). Three studies reported alternate outcomes in addition to frequency of use, with one reporting stages of initiation [39] and two studies reporting quantity of use [42], [43]. The two studies not reporting frequency of use measured new user proportion [44], [45]. On average, studies included three outcome measures (1 to 18, *Mdn* = 2). Of the 25 studies, 13 (52%) did not present post-test data, while four (16%) reported post-test data only. Thirty-six percent (*n* = 9) of trials completed follow-up within one year, while 28% (*n* = 7) had follow-up tests for two years, and 20% (*n* = 5) had follow-up periods greater than two years post program implementation.

### Data synthesis of program design

#### Universal programs

Of the set of 25 studies, 15 (60%) studies were universal prevention programs. The majority of these studies (60%, *n* = 9) utilized middle school students, with all except one [40] which recruited from community-based organizations, recruiting from a school setting. Nine (60%) of the universal programs had a significant finding (*d* = 0.08 to 5.26, *Mdn* = 0.36). The universal program conducted by Schinke and colleagues [40] obtained substantially larger effect sizes than the other universal programs (*d* = 1.63 to 5.26, *Mdn* = 2.19). When excluding this study, the median effect size of the other significant findings reduced to 0.14.

#### Targeted programs

The remaining ten studies (40%) were targeted prevention programs. These programs were targeted towards gender (*n* = 4; all female), ethnicity (*n* = 3; two African American and one Mexican American), athletic participation (*n* = 2), disease (*n* = 1; HIV positive youth), and personality risk factors (*n* = 1, high risk personality), with one study [46] covering two categories by targeting female athletes. Recruitment for participants in targeted studies utilized school settings (*n* = 5), medical clinics (*n* = 2), community organizations (*n* = 1), youth-oriented websites (*n* = 1), and radio and newspaper media advertisements (*n* = 1). Six (60%) of the targeted programs reported significant findings (*d* = 0.07 to 0.74, *Mdn* = 0.20).

#### Uni-modal programs

Uni-modal programs are those that adopted a single modality for prevention implementation. Fourteen (56%) studies utilized uni-modal program designs, of which seven (50%) were universal programs and seven (50%) were targeted. Many uni-modal programs were implemented in school settings (*n* = 9). Other delivery modes included the family home [47], community-based organizations [48], an HIV clinic [42], a computer [49], and one study delivered weekly classes in an unspecified location for participants recruited from numerous sources, including medical clinics and staff outreach [50]. Nine of the uni-modal programs reported significant findings (*d* = 0.09 to 0.74, *Mdn* = 0.20), of which four were universal (*d* = 0.09 to 0.22, *Mdn* = 0.13) and five were targeted (*d* = 0.14 to 0.74, *Mdn* = 0.20).

#### Multi-modal programs

The remaining 11 (44%) studies utilized multi-modal program designs, of which eight (73%) were universal programs and three (27%) were targeted. The primary sites for multi-modal program implementation were middle and high school settings (*n* = 9), with one study utilizing a college setting [38], and another utilizing community based organizations [40]. The core components of the multi-modal interventions involved drug prevention programs predominantly delivered through school curriculums (*n* = 7), with other programs utilizing, a CD-Rom intervention [40], a child-skills workshop [44], a motivational interviewing session [38], and a one-on-one health consultation [51]. Parent and family-based intervention components were most commonly adopted in conjunction with these core components (*n* = 8). The parent components would vary from intensive skills training workshops [44], [45], [52], [53], to take-home handbooks and information pamphlets that could be used as a basis for discussion [38], [51]. Other additional components included peer involvement (*n* = 2), community leadership/mentoring (*n* = 2), mass media coverage (*n* = 2), and school community development (*n* = 1). Of the eleven multi-modal programs, six reported significant findings (*d* = 0.07 to 5.26, *Mdn* = 0.68), of which five were universal (*d* = 0.08 to 5.26, *Mdn* = 0.90) and one was targeted (*d* = 0.07 to 0.12, *Mdn* = 0.10). When excluding the Schinke and colleagues study [40], the median effect size of the other statistically significant multi-modal study findings reduced to 0.14 and to 0.17 for the universal multi-modal study findings.

### Synthesis of individual program components

#### Participant age

Participant ages at baseline ranged from 11 to 21 years. Studies were divided into three categories to assess the optimal period of intervention: early adolescence (11 to 13 years, *n* = 14), middle adolescence (14 to 17 years, *n* = 9), and late adolescence/young adult (18+years, *n* = 2). Of those targeting early adolescence, ten (71%) programs yielded significant findings (*d* = 0.07 to 5.26, *Mdn* = 0.17), whereas only five (56%) programs targeting middle adolescence yielded significant findings (*d* = 0.14 to 0.74, *Mdn* = 0.2). No significant findings were reported for the late adolescence/young adult age group (*d* = −0.07 to 0.34, *Mdn* = 0.10, *n* = 2).

Table 5 presents the outcome data and quality ratings for participant age across universal and targeted programs, subdivided by uni-modal and multi-modal program design. Universal multi-modal programs delivered during early adolescence were associated with a median large effect size, but only a small median effect size (*d* = 0.17) when excluding the Schinke and colleagues study [40] While targeted uni-modal programs delivered during middle adolescence were associated with small effect sizes in comparison to trivial effect sizes for programs delivered during early adolescence, there were an equal number of outcome measures in this category with statistically non-significant results. For universal uni-modal and targeted multi-modal programs, no statistically significant findings yielded greater than trivial median effect sizes and these effects were not reliable.

**Table 5:** Significant and non-significant outcome data and quality ratings, as a function of program design for participant age.

	Early Adolescence					Middle Adolescence					Late Adolescence				
	*n_1_ (n_2_)*	*Mdn d*	*d* range	*Mdn* qual	Qual range	*n_1_ (n_2_)*	*Mdn d*	*d* range	*Mdn* qual	Qual range	*n_1_ (n_2_)*	*Mdn d*	*d* range	*Mdn* Qual	Qual range
**Universal uni-modal**															
Sig	3(6)	0.12	0.09–0.22	4.0	3–6	1(1)	0.14		4.0		0(0)				
Non	4(9)	0.04	−0.17–0.23	4.5	3–6	2(5)	0.11	0.09–0.17	5.0	4–6	0(0)				
**Universal multi-modal**															
Sig	5 (17)	0.90	0.08–5.26	5.0	4–8	0(0)					0(0)				
Non	3(4)	0.07	−0.03–0.10	4.0	4–5	1(3)	0.01	−0.03–0.12	3.0		0(0)				
**Targeted uni-modal**															
Sig	1(2)	0.17	0.14–0.20	8.0		4(6)	0.28	0.20–0.74	5.5	4–7	0(0)				
Non	0(0)					3(6)	0.08	−0.05–0.19	4.0	4–5	1(1)	0.34		5.0	
**Targeted multi-modal**															
Sig	1(4)	0.10	0.07–0.12	1.0		0(0)					0(0)				
Non	2(15)	−0.03	−0.07–0.30	2.0	1–3	0(0)					1(3)	0.06	−0.07–0.14	2.0	

*Note*. Sig = significant, Non = non-significant, *n_1_* = number of studies, *n_2_* = number of outcome measures, Qual = quality.

#### Program facilitators

School teachers were the principal program facilitators accounting for 33% (*n* = 11) of facilitators in the included studies. Other programs utilized health professionals (15%, *n* = 5), peer leaders (12%, *n* = 4), parents (9%, *n* = 3), project workers (9%, *n* = 3), health educators (3%, *n* = 1), community role models (3%, *n* = 1), or were self-facilitated computer programs (6%, *n* = 2), with some studies opting to use multiple facilitators for program implementation (9%, *n* = 3). Two studies were excluded from this analysis of program facilitators as they collapsed their results across groups in their statistical analyses [52]–[55], one of these studies had utilized multiple facilitators [52], [53]. As a result, only two studies assessing multiple facilitators [40], [45] could be included in the data synthesis.

Of the 23 included studies, approximately 67% (*n* = 6) of teacher-facilitated programs reported significant results (*d* = 0.07 to 0.68, *Mdn* = 0.11), compared to 37% (*n* = 7) of non-teacher facilitated programs (*d* = 0.14 to 2.86, *Mdn* = 0.51), and 100% (*n* = 2) of programs utilizing multiple leaders (*d* = 0.75 to 5.26, *Mdn* = 2.38). The non-teacher facilitated programs associated with statistically significant effect sizes were facilitated by project workers (*n* = 2), health professionals (*n* = 1), peer leaders (*n* = 1), parents (*n* = 1), or were self-facilitated computer programs (*n* = 2). The multiple facilitators programs utilized a teacher in combination with a project worker [45] or a parent in combination with a CD-Rom intervention [40].

Table 6 presents outcome data and quality ratings for program facilitators across universal and targeted programs, subdivided by uni-modal and multi-modal program design. Universal multi-modal programs were reliably associated with large effect sizes for programs utilizing non-teacher facilitators and multiple facilitators, as only one study contradicts this finding. While targeted uni-modal programs were associated with small effect sizes for programs utilizing non-teacher facilitators, there were an almost equal number of statistically non-significant findings in this category. Of those teacher facilitated programs reporting statistically significant results, no median effect size estimates were greater than trivial, and there were an equal or greater number of outcome measures with statistically non-significant results within each program design category.

**Table 6:** Significant and non-significant outcome data and quality ratings, as a function of program design for program facilitator.

	Teacher					Non-teacher					Multiple facilitators				
	*n_1_ (n_2_)*	*Mdn d*	*d* range	*Mdn* qual	Qual range	*n_1_ (n_2_)*	*Mdn d*	*d* range	*Mdn* qual	Qual range	*n_1_ (n_2_)*	*Mdn d*	*d* range	*Mdn* Qual	Qual range
**Universal uni-modal**															
Sig	3(5)	0.11	0.09–0.22	4.0	4–6	0(0)					0 (0)				
Non	3(7)	0.03	−0.17–0.23	5.0	4–6	2(5)	0.11	0.09–0.17	5.0	4–6	0(0)				
**Universal multi-modal**															
Sig	2(4)	0.12	0.08–0.68	5.5	5–6	2(6)	1.80	0.49–2.86	7.0	6–8	2(5)	2.38	0.75–5.26	7.0	6–8
Non	2(4)	0.05	−0.03–0.12	3.5	3–4	1(2)	0.02	−0.03–0.06	4.0		0(0)				
**Targeted uni-modal**															
Sig	0(0)					5(8)	0.20	0.14–0.74	6.0	5–8	0(0)				
Non	0(0)					4(7)	0.10	−0.05–0.34	4.5	4–5	0(0)				
**Targeted multi-modal**															
Sig	1(4)	0.10	0.07–0.12	1.0		0(0)					0(0)				
Non	1(14)	−0.04	−0.07–0.07	1.0		2(4)	0.10	−0.07–0.30	2.5	2–3	0(0)				

*Note*. Sig = significant, Non = non-significant, *n_1_* = number of studies, *n_2_* = number of outcome measures, Qual = quality. Two studies were excluded from this analysis of program facilitators as they collapsed their results across groups in their statistical analyses.

#### Program duration

Program duration was assessed according to the number of sessions delivered, divided into two categories: short programs (ranging from 0 to 10 sessions, *Mdn* = 5, *n* = 13) and long programs (ranging from 11 to 23 sessions, *Mdn* = 15, *n* = 12). It is important to note, however, that the length of these sessions varied from approximately 13 minutes to 90 minutes, and that the total period of time taken to implement an intervention program varied from 1 day to 3 years. Seven (54%) of the programs categorized as short (*d* = 0.07 to 5.26, *Mdn* = 0.53) and eight (67%) of the long programs (*d* = 0.08 to 0.75, *Mdn* = 0.14) yielded significant findings. Table 7 presents outcome data and quality ratings for program duration across universal and targeted programs, subdivided by uni-modal and multi-modal program design. While short universal multi-modal programs were associated with large effect sizes in comparison to trivial effect sizes for long programs, there were an equal number of short programs in this category with statistically non-significant results. Further, when removing the Schinke and colleagues study [40], the median effect size for statistically significant short universal multi-modal findings was moderate in size (*d* = 0.70) rather than large. One long targeted uni-modal program yielded a statistically significant small effect size and another obtained statistically non-significant results in this category.

**Table 7:** Significant and non-significant outcome data and quality ratings, as a function of program design for program duration.

	Short Programs					Long Programs				
	*n_1_(n_2_)*	*Mdn d*	*d* range	*Mdn* qual	Qual range	*n_1_(n_2_)*	*Mdn d*	*d* range	*Mdn* qual	Qual range
**Universal uni-modal**										
Sig	0 (0)					4 (7)	0.13	0.09 −0.22	4.0	3–6
Non	2 (5)	0.11	0.09–0.17	5.0	4–6	4 (9)	0.04	−0.17–0.23	4.5	3–6
**Universal multi-modal**										
Sig	2(10)	1.99	0.49–5.26	6.0	4–8	3 (7)	0.15	0.08–0.75	5.0	5–6
Non	2 (3)	0.06	−0.03–0.08	4.0	4–8	0 (0)				
**Targeted uni-modal**										
Sig	4 (7)	0.20	0.14–0.74	5.5	4–8	1 (1)	0.35		7.0	
Non	3 (6)	0.08	−0.05–0.34	5.0	4–5	1 (1)	0.19		4.0	
**Targeted multi-modal**										
Sig	1 (4)	0.10	0.07–0.12	1.0		0 (0)				
Non	2(17)	−0.03	−0.07–0.14	1.5	1–2	1 (1)	0.30		3.0	

*Note*. Sig = significant, Non = non-significant, *n_1_* = number of studies, *n_2_* = number of outcome measures, Qual = quality.

#### Booster sessions

Booster sessions were often implemented in addition to the core program sessions. The number of booster sessions ranged from 1 to 15, with specific information on session length, time of implementation, and content often not reported. For these reasons, studies were divided into two categories, those that implemented boosters (*n* = 9) and those that did not (*n* = 16). Eighty-nine percent of programs that utilized boosters reported significant findings (*d* = 0.07 to 5.26, *Mdn* = 0.15, *n* = 8), in contrast to only 44% of programs that did not utilize boosters (*d* = 0.14 to 0.90, *Mdn* = 0.22, *n* = 7). Table 8 presents the outcome data and quality ratings for the presence of booster sessions across universal and targeted programs, subdivided by uni-modal and multi-modal program design. Unlike other program components, studies with booster sessions were more often associated with statistical significance than studies without booster sessions; however, the effects were often trivial or small. Only two universal multi-modal programs were associated with moderate to large effect sizes, and one of these studies was the Schinke and colleagues study [40]. Programs without boosters more often reported non-significant findings, and when statistically significant findings were reported, their median effect size was small or trivial.

**Table 8:** Significant and non-significant outcome data and quality ratings, as a function of program design for booster sessions.

	With boosters					Without boosters				
	*n_1_(n_2_)*	*Mdn d*	*d* range	*Mdn* qual	Qual range	*n_1_(n_2_)*	*Mdn d*	*d* range	*Mdn* qual	Qual range
**Universal uni-modal**										
Sig	2 (5)	0.11	0.09–0.13	3.5	3–4	2 (2)	0.18	0.14–0.22	5.0	4–6
Non	3 (7)	0.04	0.01–0.11	4.0	3–5	3 (7)	0.11	−0.17–0.23	6.0	4–6
**Universal multi-modal**										
Sig	3(13)	1.96	0.08–5.26	6.0	5–8	2 (4)	0.33	0.14–0.90	4.5	4–5
Non	0 (0)					4 (7)	0.06	−0.03–0.12	4.0	3–5
**Targeted uni-modal**										
Sig	2 (3)	0.20	0.14–0.20	6.5	5–8	3 (5)	0.35	0.20–0.74	6.0	4–7
Non	1 (3)	0.10	−0.04–0.12	5.0		3 (4)	0.13	−0.05–0.34	4.0	4–5
**Targeted multi-modal**										
Sig	1 (4)	0.10	0.07–0.12	1.0		0 (0)				
Non	1(14)	−0.04	−0.07–0.07	1.0		2 (4)	0.10	−0.07–0.30	2.5	2–3

*Note.* Sig = significant, Non = non-significant, *n_1_* = number of studies, *n_2_* = number of outcome measures, Qual = quality.

### Evaluation of study quality

The two independent raters agreed on 97% of the quality scores. After discrepancies were resolved, quality scores ranged from 1 to 8, with an average quality rating of 4.64. This indicates that for many of of studies, half of the quality criteria were either not met. Quality ratings were consistent across universal uni-modal (*Mdn* = 4.0, range 3–6), universal multi-modal (*Mdn* = 4.5, range 3–8), and targeted uni-modal (*Mdn* = 5.0, range 4–8) program designs, with lower quality reported for targeted multi-modal programs (*Mdn* = 2, range 1–3). The two highest quality studies were both conducted by Schinke and colleagues [40], [47]. Their universal multi-modal study produced the largest effect sizes examined in this review, while their targeted uni-modal study was associated with trivial to small effects.

Overall, irrespective of program design, most studies scored positively on criteria assessing baseline outcomes (92%) and baseline characteristics (84%). A moderate percentage of studies scored positively on criteria assessing contamination (64%) and selective reporting (72%). In contrast, only half of all studies (52%) scored positively on the incomplete data criterion. The poor scores for incomplete data were a result of extremely high attrition rates, poor statistical treatment of missing data, or a lack of reporting. Of those studies scoring zero for poor attrition, attrition rates ranged from 23% to 47%, for follow-up periods from immediate post-test to 6 years.

Quality scores were particularly problematic on criteria assessing program exposure, program adherence, blinding, and reliability of outcome measure with only 20%, 20%, 24%, and 40% of studies, respectively, scoring positively on these measures. While these scores could simply be a result of poor reporting, not being able to adequately assess these methodological components prevents a meaningful analysis of program curriculum.

## Discussion

This systematic review evaluated the effectiveness of primary prevention programs in averting young people from using cannabis. The current study extended knowledge gained from previous reviews by assessing the relative efficacy of universal, targeted, uni-modal, and multi-modal approaches, and evaluating whether the effectiveness of individual program components was related to program type. Overall, results suggest that primary prevention programs may be able to deter young people from using cannabis, with statistically significant effect sizes ranging from trivial (0.07) to extremely large (5.26). Despite this potential, evidence was largely inconclusive regarding a distinctive pattern of program efficacy as the percentages of statistically significant and non-statistically significant findings were often equivalent across program type and individual components. A consideration of the magnitude of statistically significant median effect size estimates, however, did reveal components that may more strongly influence program efficacy. The efficacy of these components must be interpreted with caution as they may be unreliable and biased given the equivalency of significant and non-significant data across categories, as well as the variability of study quality. Furthermore, a visual inspection of the data indicated that one study [40] had dramatically larger effect size values than all other included studies (*d* range = 1.63 to 5.26), which may have overestimated the magnitude of median effect sizes. As such, results for categories where this study was included must be interpreted with caution as analysis without this study included often resulted in substantially smaller effects.

### Cannabis specific-content

Most of the of studies included in this review did not include cannabis-specific content, hindering efforts to make conclusive statements about the relative efficacy of cannabis-specific versus general substance use prevention programming. Despite this, some programs were able to effectively prevent cannabis use for at least a short period. Tobler et al. [24] found that programs that were effective in preventing cannabis use were similarly effective for alcohol and tobacco use. As such, they suggested that cannabis did not require a singularly focused program. Similarly, Foxcroft et al. [56] found no apparent differences in program efficacy in an assessment of alcohol specific versus multi-drug prevention programming. Given that some programs included in the current review were able to avert cannabis use, despite not exclusively focusing on cannabis-related content, it may be that specific content is not a necessary component for program efficacy. In fact, Tobler et al. [24] suggested that the relative effectiveness of a program was dependent on delivery method, not program content. Research that specifically examines the relative efficacy of a singularly focused cannabis-use prevention program versus a multi-drug prevention program is needed to elucidate these suggestions.

### Program type

Prior to this study, scarce research examining the relative efficacy of universal, targeted, uni-modal, and multi-modal programs existed, with the preponderance of cannabis-specific and general substance use prevention reviews and meta-analyses assessing universal school-based (uni-modal) approaches only. Overall, a consideration of statistically significant median effect size magnitudes revealed that universal multi-modal programs may be more effective in averting cannabis use than universal uni-modal, targeted uni-modal, and targeted multi-modal programs. Importantly, all three targeted multi-modal program studies were of poor quality, thus results pertaining to this category may be unreliable and should be interpreted with caution. Furthermore, the effectiveness of universal multi-modal studies appeared to be much greater when the study by Schinke and colleagues was included (*Mdn d* = 0.90 versus 0.17). As this study was of better quality than the others, universal multi-modal programs may be quite effective in preventing cannabis use when they are adopted and implemented as intended.

The finding that universal multi-modal programs may outperform universal uni-modal, targeted uni-modal, and targeted multi-modal programs implies that the combination of multi-modal and universal strategies may be particularly important. There is growing evidence which suggests that multi-modal prevention strategies improve effectiveness [27], , a finding which is consistent with problem-behavior theory that espouses the importance of concurrently targeting multiple domains [6], [58]. Thus, it is not surprising that multi-modal programs appear to outperform uni-modal programs. The fact that this effect was only evident for universal programs, however, is somewhat puzzlingly given that the limited evidence available implicates the potential efficacy of both universal and targeted approaches [59]. It may be that utilizing a multi-modal strategy which facilitates a broad-spectrum approach is necessary for universal programs which endeavor to reach a widely varied population. Conversely, targeted programs which are designed to address an identified high-risk population may not similarly necessitate a multi-modal approach and perhaps a multi-modal strategy, in targeting multiple domains, may actually dilute and offset important messages. Alternatively, it may be that the poor quality of the targeted multi-modal programs resulted in an underestimation of efficacy or that targeted programs overall are not effective. Given that most of the research to date had focused on universal programming, future research is needed which specifically addresses targeted approaches.

### Individual program components

#### Participant age

Overall, programs targeting early and middle adolescence may yield small beneficial effects, whereas programs targeting late adolescence may not be effective, as no statistically significant results were reported. Given that the estimated age of initiation for cannabis use is between 15.9 and 18.4 years [60], [61], and that early initiation is associated with an increased risk for problematic outcomes, it may be that utilizing primary prevention strategies for late adolescence (greater than 18 years) is too late to avert use. The substance use literature has predominantly reported inconclusive findings regarding the optimal developmental stage for program delivery [17], [20]. An analysis of participant age as a function of program type revealed that universal multi-modal programs may benefit from addressing early adolescence, whereas targeted uni-modal programs may be more effective when targeting middle adolescence, though this result for the latter finding was not reliable. Future research should directly examine the relative efficacy of cannabis prevention delivered during early and middle adolescence.

#### Program facilitator

Consistent with the general substance use [17], [21] and cannabis-specific [25] prevention literature, programs utilizing non-teacher facilitators or multiple facilitators may be more effective than programs utilizing teachers only, when considering the magnitude of statistically significant median effect sizes. This finding was evident only for universal multi-modal programs, with either statistically non-significant or unreliable outcome data reported across other categories. Importantly, an assessment of studies reporting statistical significance indicated that programs utilizing teachers and multiple facilitators were more likely to yield statistically significant outcomes than programs utilizing non-teacher facilitators. These marked discrepancies in patterns of results highlight the importance of conducting a holistic assessment of both statistical and clinical significance. Insufficient data was available to further specify which non-teacher facilitators and which combination of multiple facilitators were more effective, as a maximum of only two studies reporting statistically significant effect sizes for non-teacher facilitators and multiple leaders per category were reported. Future research is needed to elucidate these findings.

#### Program duration

Inconsistent with previous cannabis-specific research [25], the results of the current study suggest that programs shorter in duration may be more efficacious than longer programs. On the whole, results pertaining to program duration largely have been inconclusive [20], [62]. Tobler et al. [17], however, found that while program efficacy was not related to program duration overall, a re-analysis of duration as a function of program type (i.e., interactive or non-interactive) revealed that interactive programs benefited from longer duration, a benefit which was not evident for non-interactive programs. In accordance, the results of the current study revealed apparent differences in program efficacy as a function of program type; specifically, universal multi-modal programs may be more effective when shorter in duration, whereas targeted uni-modal programs may benefit from longer duration, though this latter result was not reliable. Further research is needed to confirm the relative efficacy of program duration (both time period and number of sessions) as a function of program type.

#### Boosters

The majority of studies utilizing booster sessions yielded statistically significant results. An assessment of the magnitude of statistically significant median effect size data, however, revealed that only universal multi-modal programs were reliably associated with effective outcomes when boosters were implemented. While this finding may be surprising given that previous literature has consistently found that booster sessions are associated with both increasing and maintaining program efficacy [23], [63], it may be that the effectiveness of boosters is intrinsically linked to and may be dependent on aspects of program design (e.g., content or format; [62]). Gottfredson and Wilson [59] suggest that conclusions pertaining to booster sessions are largely drawn from a handful of instructional programs, presenting analogous program designs (e.g., LST) [54], thus future research should continue to assess the relative efficacy of implementing boosters for a variety of program designs. In addition, further research is needed to ascertain the specific details (e.g., length, time of implementation, and content) of effective booster sessions, an assessment that was beyond the scope of the current review due to poor reporting.

### Quality of studies

Despite adopting rigorous inclusion criteria such that only RCTs published in peer-reviewed English journals were eligible for inclusion, on the whole, the quality of included studies was quite poor. Problems of poor quality were related to high levels of attrition and missing data, the use of inadequate outcome measures, inadequate reporting of program components and implementation fidelity, as well as a general poor reporting of salient methodological features (e.g., methods of blinding and baseline cannabis use outcomes). While high levels of attrition, in general, cause a serious threat to internal and external validity [64], in the current review levels of attrition were particularly problematic as they often were differentially related to baseline cannabis use. While some studies applied statistical methods to reduce the bias caused by differential attrition (e.g., intent to treat analysis, inclusion of baseline cannabis use as covariate) future research should focus on maintaining retention throughout follow-up periods [65], [66]. In addition, included studies often failed to report or assess implementation fidelity (i.e., program exposure and program adherence), rendering an assessment of specific program curriculum components meaningless. High implementation fidelity has been highlighted as a key feature to achieving program effectiveness [67]; thus, reporting implementation fidelity in prevention trails is necessary to facilitate an adequate and appropriate assessment of program efficacy.

Marked variability of statistical methodology, cannabis use outcomes, and follow-up periods across studies, rendered a meaningful meta-analysis untenable. Future research may benefit from the development of a standardized procedure for assessing cannabis use which stipulates the use of psychometrically verified assessments as well as a directive timeline for appropriate follow-up intervals. In addition, studies often failed to report important statistical information (e.g., group sample size, standard deviations, mean scores), which is required to calculate effect size and confidence interval values. Solely presenting traditional null hypothesis testing is no longer considered sufficient as it only provides information regarding statistical significance and does not facilitate a meaningful assessment of clinical or practical significance.

### Implications for Research

Future research must become more rigorous in reporting important methodological program characteristics (e.g., program content, delivery mode, and program fidelity) and salient statistical information (e.g., means and standard deviations, sample size values, and problems with attrition). The development of such procedures will not only enable a quantitative assessment of outcome data, which was not feasible in the current review, but will also facilitate a more rigorous assessment of all program components. In addition, results of the current study implicate the importance of assessing the inter-dependent relationship between individual program components and program type. Specifically, individual program components appeared to effect program efficacy as a function of program design. Thus, it seems pertinent that future research acknowledges the inter-dependent nature of different elements of program design and modifies assessment procedures to reflect these relationships. Furthermore, future research should evaluate the potential efficacy of universal multi-modal designs, targeting early adolescence, short in duration, implementing boosters, and utilizing non-teacher facilitators, rather than simply continuing to disseminate existing programs.

In addition, research is needed to assess theoretical foundations for effective program development and to identify specific elements of effective program content and design. Prior research in the broader substance use literature [18]–[20] has often dichotomized information on program theory (e.g., psychosocial and non-psychosocial), program content (e.g., affective vs. knowledge-based), and program design (e.g., interactive and non-interactive) to provide a basic understanding of effective components. While these categorizations are informative, they do not provide sufficient or holistic information to enable specific developments in program components or allow for program replication. If the quality of program reporting improves a more rigorous assessment of effective theory-driven components, content areas (e.g., interpersonal skills, intrapersonal development, and substance use knowledge) and delivery methods (e.g., discussions, role plays, and computer activities) may be feasible. As the word limit of certain scientific journals may not allow for sufficient reporting a more rigorous examination of program manuals is required or authors need to focus on submitting to journals without strict word limits. Further research is also needed to substantiate suggestions that cannabis-specific content may not provide any additional benefit to effectively prevent cannabis-use, over and above general substance use prevention strategies. In addition, the long-term follow-up of prevention studies needs to extend past high school. In order to determine if primary prevention programs are effective in reducing harms, not just use, research need to examine how long programs can delay use for and whether or not programs universally prevent cannabis use or if they only work for those who would have become infrequent users. Finally, as the preponderance of studies included in this review were conducted in the United States, the above recommendations for future prevention research need to be carried out in a variety of different countries. Prevention programs that work in the United States may not be effective in countries or cultures that have different rates of teenage cannabis use or different views on how and when cannabis use should be discussed. For example, educational systems in countries other than the United States may prohibit drug prevention programs from being delivered during early adolescence. Cultural modifications need to be examined for their efficacy before policymakers adopt a specific program.

### Implications for Policy

Primary prevention strategies are often considered the most valuable approaches for targeting substance use as they can be extremely cost-effective [10], [68] and have an enormous capacity to prevent substance use across a wide range of individuals. Thus, it is imperative that dissemination of primary prevention is consistent with the scientific literature. Given that reliable and clearly discernible patterns of program efficacy remain largely inconclusive, further research is needed to elucidate effective prevention strategies. At this point in time, policymakers should be concerned with allotting money for high quality primary prevention research studies with long-term follow-up that continues beyond high school. Although this review has highlighted that some programs work, their effects often are trivial to small over a few years, and often they are not adopted or implemented as intended. If programs cannot be carried out with high fidelity during the research process, it is likely they also will be poorly adopted and implemented once mandated. The two studies that achieved the highest quality ratings in this review examined programs delivered by a computer. Given the growing use of technology, and the ability of computerized interventions to be delivered as intended, policymakers should strongly consider their use for program delivery. Further, policymakers need to strongly consider the potential benefits of programs that target early adolescence, are short in duration, utilize booster sessions and non-teacher facilitator rather than simply disseminating programs that may appeal to educational systems or concerned adults. After all, it is the children for whom the programs are intended. Lastly, policymakers need to consider that when programs work, they may only work minimally, and additional consideration of secondary prevention programs may be necessary.

### Strengths and limitations

The current review extended on previous literature by providing a broad-spectrum approach to assessing primary prevention program efficacy. The inclusion of universal and targeted programs, uni-modal and multi-modal strategies, in addition to the assessment of individual program components, facilitated a comprehensive synthesis of existing primary prevention strategies. In addition, given that cannabis is the most widely used illicit drug worldwide [1], [69] and that individually focused reviews of cannabis prevention are scarce, the specific focus on cannabis use adopted in this review provided much needed knowledge and direction. Furthermore, including quality ratings as well as presenting both significant and non-significant outcome data provides an unbiased and comprehensive synthesis of included studies.

Nevertheless, there are several limitations that must be considered. First, the inclusion of only peer-reviewed published articles may have inadvertently biased prevention results, as studies reporting statistically significant findings are more likely to be published [70]. The inclusion of grey literature, in addition to published literature, may provide a more wide-ranging overview of available programs. Second, an appropriate meta-analysis was not feasible due to the marked heterogeneity of study design and measurement outcomes. As a result, a narrative review that synthesized a wide-variety of approaches was utilized. The diversity within a prevention strategy (e.g., different delivery modalities for universal uni-modal programs) may have obscured important findings. Third, multiple effect size values were often reported for an individual study and the inconsistency of the magnitude of reported effect size values across studies may have skewed results. An improvement of methodological and reporting quality of prevention trials would facilitate an appropriate quantitative analysis. Fourth, the individual program components assessed in this study also were limited due to poor reporting of salient and important information. All included studies relied on self-reported cannabis use, which may not provide an accurate measure of cannabis use; however, evidence has supported the validity of such self-reported data [71]. Lastly, 21 of the 49 articles that met inclusion criteria were excluded due to the unavailability of data. If data were available, inclusion of these studies may have resulted in vastly different outcomes.

## Conclusions

Overall, the current study suggests primary prevention programming may avert cannabis use. Albeit reliable and discernible patterns for program efficacy remain largely inconclusive, results of the current study implicate the importance of assessing the relative efficacy of all program types and the inter-dependent relationship of program type and individual program components. Substantial work is needed to improve the methodological and statistical reporting quality of effectiveness trials. The improvement of study quality in addition to continued research developing new models of prevention programs that consider the inter-dependent nature of individual program components will enable a successful approach to preventing cannabis use. Given the high prevalence of cannabis use in young people [2], [3] and the extent of problems associated with early initiation [6], [7], further developments in this area are pertinent.

## Supporting Information

Appendix S1 **Search strategy for CINAHL (Ovid) 1987–2011.** Limits included English language, peer reviewed and human.
(DOCX)

Appendix S2 **Quality Criteria (adapted from EPOC Risk of Bias).**
(DOCX)

Appendix S3 **List of excluded eligible studies (n = 21).**
(DOCX)

## References

[1] UNODC (2011) World Drug Report 2011. Vienna: United Nations Office on Drugs and Crime.

[2] Degenhardt L, Chiu W-T, Sampson N, Kessler RC, Anthony JC, et al (2010) Towards a global view of alcohol, tobacco, cannabis, and cocaine use: Findings from the WHO world mental health surveys. PLoS Med 5: 1053–1067. 10.1371/journal.pmed.0050141 PMC2443200 18597549

[3] AIHW (2011) 2010 National Drug Strategy Household Survey Report. Canberra: Australian Institute of Health and Welfare.

[4] Mayet A, Legleye S, Chau N, Falissard B (2010) The mediation role of licit drugs in the influence of socializing on cannabis use among adolescents: A quantitative approach. Addict Behav 35: 890–895. 20584572 10.1016/j.addbeh.2010.06.001

[5] Von Sydow K, Lieb R, Pfister H, Hofler M, Wittchen H-U (2002) What predicts incident use of cannabis and progression to abuse and dependence? Drug Alcohol Depend 68: 49–64. 12167552 10.1016/s0376-8716(02)00102-3

[6] DuRant RH, Smith JA, Kreiter SR, Krowchuk DP (1999) The relationship between early age of onset of initial substance use and engaging in multiple health risk behaviors among young adolescents. Arch Pediatr Adolesc Med 153: 286–291. 10086407 10.1001/archpedi.153.3.286

[7] Fergusson DM, Horwood LJ, Swain-Campbell N (2002) Cannabis use and psychosocial adjustment in adolescence and young adulthood. Addiction 97: 1123–1135. 12199828 10.1046/j.1360-0443.2002.00103.x

[8] Ellickson PL, Tucker JS, Klein DJ, Saner H (2004) Antecedents and outcomes of marijuana use initiation during adolescence. Prev Med 39: 976–984. 15475032 10.1016/j.ypmed.2004.04.013

[9] Fergusson DM, Boden JM (2008) Cannabis use and later life outcomes. Addiction 103: 969–976. 18482420 10.1111/j.1360-0443.2008.02221.x

[10] Sanderson CA (2004) Health Psychology. Hoboken: John Wiley & Sons Inc.

[11] Dragt S, Nieman DH, Becker HE, Van de Fliert R, Dingemans PM, et al (2010) Age of onset of cannabis use is associated with age of onset of high-risk symptoms for psychosis. Can J Psychiatry 55: 165–171. 20370967 10.1177/070674371005500308

[12] De Graaf R, Rodavanovic M, van Laar M, Fairman B, Degenhardt L, et al (2010) Early cannabis use and estimated risk of later onset of depression spells: Epidemiologic evidence from the population-based world health organisation world mental health survey initiative. Am J Epidemiol 172: 149–159. 20534820 10.1093/aje/kwq096 PMC2915487

[13] AIHW (2008) 2007 National Drug Strategy Household Survey. Canberra: Australian Institute of Health and Welfare.

[14] Agosti V, Levin FR (2004) Predictors of treatment contact among individuals with cannabis dependence. Am J Drug Alcohol Abuse 30: 121–127. 15083557 10.1081/ada-120029869

[15] Stephens RS, Roffman RA, Simpson EE (1994) Treating adult marijuana dependence: a test of the relapse prevention model. J Consult Clin Psychol 62: 92–99. 8034835 10.1037//0022-006x.62.1.92

[16] Carroll KM, Easton CJ, Nich C, Hunkele KA, Neavins TM, et al (2006) The use of contingency management and motivational/skills-building therapy to treat young adults with marijuana dependence. J Consult Clin Psychol 74: 955–966. 17032099 10.1037/0022-006X.74.5.955 PMC2148500

[17] Tobler NS, Roona M, Ochshorn P, Marshall DG, Streke AV, et al (2000) School-based adolescent drug prevention programs: 1998 meta-analysis. J Prim Prev 20: 275–336.

[18] Faggiano F, Vigna-Taglianti FD, Versino EZ, Borraccino A, Lemma P (2008) School-based prevention for illicit drugs use: A systematic review. Prev Med 46: 385–396. 18258289 10.1016/j.ypmed.2007.11.012

[19] Tobler NS (1986) Meta-analysis of 143 adolescent drug prevention programs: Quantitative outcome results of program participants compared to a control or comparison group. J Drug Issues 16: 537–567.

[20] Tobler NS, Stratton HH (1997) Effectiveness of school-based drug prevention programs: A meta-analysis of the research. J Prim Prev 18: 71–128.

[21] Tobler NS (1992) Drug prevention programs can work. J Addict Dis 11: 1–28. 10.1300/J069v11n03_01 1320942

[22] Gates S, McCambridge J, Smith LA, Foxcroft D (2006) Interventions for prevention of drug use by young people delivered in non-school settings (Review). Cochrane Database Syst Rev 10.1002/14651858.CD005030.pub2 PMC13222737 16437511

[23] White D, Pitts M (1998) Educating young people about drugs: a systematic review. Addiction 93: 1475–1487. 9926552 10.1046/j.1360-0443.1998.931014754.x

[24] Tobler NS, Lessard T, Marshall D, Ochshorn P, Roona M (1999) Effectiveness of school-based drug prevention programs for marijuana use. Sch Psychol Int 20: 105–137.

[25] Porath-Waller AJ, Beasley E, Beirness DJ (2010) A meta-analytic review of school-based prevention for cannabis use. Health Educ Behav 37: 709–723. 20522782 10.1177/1090198110361315

[26] Lemstra M, Bennett N, Nannapaneni U, Neudorf C, Warren L, et al (2010) A systematic review of school-based marijuana and alcohol prevention programs targeting adolescents aged 10–15. Addict Res Theory 18: 84–96.

[27] Nation M, Crusto C, Wandersman A, Kumpfer KL, Seybolt D, et al (2003) What works in prevention: principles of effective prevention programs. Am Psychol 58: 449–456. 12971191 10.1037/0003-066x.58.6-7.449

[28] Gorman DM (1992) Using theory and basic research to target primary prevention programs: recent developments and future prospects. Alcohol Alcohol 27: 583–594. 1292431

[29] Griffin KW, Botvin GJ, Nichols TR, Doyle MM (2003) Effectiveness of a universal drug abuse prevention approach for youth at high risk for substance use initiation. Prev Med 36: 1–7. 12473419 10.1006/pmed.2002.1133

[30] Collaboration C (2011) Cochrane Handbook for Systematic Reviews of Interventions. In: Higgins JP, Green S, editors. Cochrane Handbook for Systematic Reviews of Interventions. 5.1.0 ed: The Cochrane Collaboration.

[31] Crombie IK, Davies HT (2009) What is a meta-analysis? Evidence-based medicine: What is? series. UK: Hayward Medical Communications.

[32] EPOC (2009) Data Collection Checklist. 64 Risk of Bias: Cochrane Effective Practice and Organisation of Care Review Group.

[33] Cohen J (1988) Statistical power analysis for the beahvioral sciences. Hillsdale, NJ: Erlbaum.

[34] Nakagawa S, Cuthill IC (2007) Effect size, confidence interval and statistical significance: a practical guide for biologists. Biol Rev Camb Philos Soc 82: 591–605. 17944619 10.1111/j.1469-185X.2007.00027.x

[35] Rosenthal R, Rubin DB (1986) Meta-analytic procedures for combining studies with multiple effect sizes. Psychol Bull 99: 400–406.

[36] Wilson DB (2001) Practical meta-analysis effect size calculator.

[37] Chinn S (2000) A simple method for converting an odds ratio to effect size for use in meta-analysis. Stat Med 19: 3127–3131. 11113947 10.1002/1097-0258(20001130)19:22<3127::aid-sim784>3.0.co;2-m

[38] Grossbard JR, Mastroleo NR, Kilmer JR, Lee CM, Turrisi R, et al (2010) Substance use patterns among first-year college students: secondary effects of a combined alcohol intervention. J Subst Abuse Treat 39: 384–390. 20817383 10.1016/j.jsat.2010.07.001 PMC2967640

[39] Werch C, Moore MJ, DiClemente CC, Bledsoe R, Jobli E (2005) A multihealth behavior intervention integrating physical activity and substance use prevention for adolescents. Prevention Science: The Official Journal of the Society for Prevention Research 6: 213–226. 16133900 10.1007/s11121-005-0012-3

[40] Schinke SP, Schwinn TM, Di Noia J, Cole KC (2004) Reducing the risks of alcohol use among urban youth: three-year effects of a computer-based intervention with and without parent involvement. J Stud Alcohol 65: 443–449. 15376818 10.15288/jsa.2004.65.443 PMC2795165

[41] Newton NC, Teesson M, Vogl LE, Andrews G (2010) Internet-based prevention for alcohol and cannabis use: final results of the climate schools course. Addiction 105: 749–759. 20148791 10.1111/j.1360-0443.2009.02853.x

[42] Naar-King S, Wright K, Parsons JT, Frey M, Templin T, et al (2006) Healthy choices: motivational enhancement therapy for health risk behaviors in HIV-positive youth. AIDS Educ Prev 18: 1–11. 16539572 10.1521/aeap.2006.18.1.1

[43] Hecht ML, Marsiglia FF, Elek E, Wagstaff DA, Kulis S, et al (2003) Culturally grounded substance use prevention: an evaluation of the keepin' it R.E.A.L. curriculum. Prevention Science: The Official Journal of the Society for Prevention Research 4: 233–248. 14598996 10.1023/a:1026016131401

[44] Spoth RL, Redmond C, Shin C (2001) Randomized trial of brief family interventions for general populations: adolescent substance use outcomes 4 years following baseline. J Consult Clin Psychol 69: 627–642. 11550729 10.1037//0022-006x.69.4.627

[45] Spoth RL, Redmond C, Trudeau L, Shin C (2002) Longitudinal substance initiation outcomes for a universal preventive intervention combining family and school programs. Psychol Addict Behav 16: 129–134. 12079251

[46] Elliot DL, Goldberg L, Moe EL, DeFrancesco CA, Durham MB, et al (2008) Long-term outcomes of the ATHENA (Athletes Targeting Healthy Exercise and Nutrition Alternatives) Program for female high school athletes. Journal of Alcohol and Drug Education 52: 73–92. 19081833 PMC2598770

[47] Schinke SP, Fang L, Cole KC (2009) Computer-delivered, parent-involvement intervention to prevent substance use among adolescent girls. Prev Med 49: 429–435. 19682490 10.1016/j.ypmed.2009.08.001 PMC2783411

[48] Stanton B, Cole M, Galbraith J, Li X, Pendleton S, et al (2004) Randomized trial of a parent intervention. Arch Pediatr Adolesc Med 158. 10.1001/archpedi.158.10.947 15466681

[49] Schwinn TM, Schinke SP, Di Noia J (2010) Preventing drug abuse among adolescent girls: outcome data from an internet-based intervention. Prev Sci 11: 24–32. 19728091 10.1007/s11121-009-0146-9 PMC2822104

[50] Palinkas LA, Atkins CJ, Miller C, Ferreira D (1996) Social skills training for drug prevention in high-risk female adolescents. Prev Med 25: 692–701. 8936571 10.1006/pmed.1996.0108

[51] Werch C, Moore MM, DiClemente CC, Owen DM, Carlson JM, et al (2005) Single vs. multiple drug prevention: is more always better?: A pilot study. Subst Use Misuse 40: 1085–1101. 16040370 10.1081/JA-200030814

[52] Faggiano F, Galanti MR, Bohrn K, Burkhart G, Vigna-Taglianti F, et al (2008) The effectiveness of a school-based substance abuse prevention program: EU-Dap cluster randomised controlled trial. Prev Med 47: 537–543. 18657569 10.1016/j.ypmed.2008.06.018

[53] Faggiano F, Vigna-Taglianti F, Burkhart G, Bohrn K, Cuomo L, et al (2010) The effectiveness of a school-based substance abuse prevention program: 18-month follow-up of the EU-Dap cluster randomized controlled trial. Drug Alcohol Depend 108: 56–64. 20080363 10.1016/j.drugalcdep.2009.11.018

[54] Botvin GJ, Baker E, Renick NL, Filazzola AD, Botvin EM (1984) A cognitive-behavioral approach to substance abuse prevention. Addict Behav 9: 137–147. 6611026 10.1016/0306-4603(84)90051-0

[55] Botvin GJ, Baker E, Filazzola AD, Botvin EM (1990) A cognitive-behavioral approach to substance abuse prevention: one-year follow-up. Addict Behav 15: 47–63. 2316411 10.1016/0306-4603(90)90006-j

[56] Foxcroft DR, Ireland D, Lister-Sharp DJL, Breen R (2003) Longer-term primary prevention for alcohol misuse in young people: a systematic review. Addiction 98. 10.1046/j.1360-0443.2003.00355.x 12653810

[57] Coie JD, Watt NF, West SG, Hawkins JD, Asarnow JR, et al (1993) The science of prevention: a conceptual framework and some directions for a national research program. Am Psychol 48: 1013–1022. 8256874 10.1037//0003-066x.48.10.1013

[58] Jessor R (1992) Risk behaviour in adolescence: a psychosocial framework for understanding and action. Dev Rev 12: 374–390. 10.1016/1054-139x(91)90007-k 1799569

[59] Gottfredson DC, Wilson DB (2003) Characteristics of effective school-based substance abuse prevention. Prev Sci 4: 27–38. 12611417 10.1023/a:1021782710278

[60] AIHW (2011) Young Australians: Their Health and Wellbeing 2011. Canberra: Australian Institute of Health and Welfare 2011.

[61] SAMHSA (2011) Results from the 2010 National Survey on drug use and health: Summary of National Findings. Rockville, Maryland: Substance Abuse and Mental Health Services Administration.

[62] Cuijpers P (2002) Effective ingredients of school-based drug prevention programs: a systematic review. Addict Behav 27: 1009–1023. 12369469 10.1016/s0306-4603(02)00295-2

[63] Botvin GJ, Renick NL, Baker E (1983) The effects of scheduling format and booster sessions on a broad-spectrum psychosocial approach to smoking prevention. J Behav Med 6: 359–379. 6668603 10.1007/BF00846324

[64] Hansen WB, Tobler NS, Graham JW (1990) Attrition in substance abuse prevention research: a meta-analysis of 85 longitudinally followed cohorts. Eval Rev 14: 667–685.

[65] Scott CK (2004) A replicable model for achieving over 90% follow-up rates in longitudinal studies of substance abusers. Drug Alcohol Depend 74: 21–36. 15072804 10.1016/j.drugalcdep.2003.11.007 PMC5937263

[66] Marmor JK, Oliveria SA, Donahue RP, Garrahie EJ, White MJ, et al (1991) Factors encouraging cohort maintenance in a longitudinal study. J Clin Epidemiol 44: 531–535. 2037857 10.1016/0895-4356(91)90216-v

[67] Griffin KW, Mahadeo M, Weinstein J, Botvin G (2006) Program implementation fidelity and substance use outcomes among middle school students in a drug abuse prevention program. Salud Drogas 6: 9–28.

[68] Caltabiano ML, Sarafino EP, Byrne D (2008) Health Psychology: Biopsychosocial interactions. Milton: John Wiley & Sons Australia, Ltd.

[69] UN (2003) World Youth Report 2003. New York: United Nations.

[70] Dwan K, Altman DG, Arnaiz JA, Bloom J, Chan A-W, et al (2008) Systemtic review of the empirical evidence of study publication bias and outcome reporting bias. PLoS One 3: 1–31. 10.1371/journal.pone.0003081 PMC2518111 18769481

[71] O'Malley PM, Bachman JG, Johnston LD (1983) Reliability and consistency in self-reports of drug use. Int J Addict 18: 805–824. 6605313 10.3109/10826088309033049

[72] Botvin GJ, Baker E, Dusenbury L, Botvin EM, Diaz T (1995) Long-term follow-up results of a randomized drug abuse prevention trial in a white middle-class population. JAMA 273: 1106–1112. 7707598

[73] Botvin GJ, Baker E, Dusenbury L, Tortu S, Botvin EM (1990) Preventing adolescent drug abuse through a multimodal cognitive-behavioral approach: results of a 3-year study. J Consult Clin Psychol 58: 437–446. 2212181 10.1037//0022-006x.58.4.437

[74] Dent CW, Sussman S, Stacy AM (2001) Project towards no drug abuse: generalizability to a general high school sample. Prev Med 32: 514–520. 11394955 10.1006/pmed.2001.0834

[75] Ringwalt CL, Clark HK, Hanley S, Shamblen SR, Flewelling RL (2009) Project ALERT: A cluster randomized trial. Arch Pediatr Adolesc Med 163: 625–632. 19581545 10.1001/archpediatrics.2009.88

[76] Rohrbach LA, Sun P, Sussman S (2010) One-year follow-up evaluation of the Project Towards No Drug Abuse (TND) dissemination trial. Prev Med 51: 313–319. 20655946 10.1016/j.ypmed.2010.07.016 PMC2939247

[77] Bond L, Patton G, Glover S, Carlin JB, Butler H, et al (2004) The Gatehouse Project: can a multilevel school intervention affect emotional wellbeing and health risk behaviors? J Epidemiol Community Health 58: 997–1003. 15547059 10.1136/jech.2003.009449 PMC1732649

[78] Ellickson PL, McCaffrey DF, Ghosh-Dastidar B, Longshore DL (2003) New inroads in preventing adolescent drug use: results from a large-scale trial of Project ALERT in middle schools. Am J Public Health 93: 1830–1836. 14600049 10.2105/ajph.93.11.1830 PMC1448059

[79] Johnson CA, Pentz MA, Weber MD, Dwyer JH, Baer N, et al (1990) Relative effectiveness of comprehensive community programming for drug abuse rpevention with high-risk and low-risk adolescents. J Consult Clin Psychol 58: 447–456. 2212182 10.1037//0022-006x.58.4.447

[80] Spoth RL, Redmond C, Trudeau L, Shin C (2002) Longitudinal Substance Initiation Outcomes for a universal preventive intervention combining family and school programs. Psychology of Addictive Behaviors 16: 129–134. 12079251

[81] Conrod PJ, Castellanos-Ryan N, Strang J (2010) Brief, personality-targeted coping skills interventions and survival as a non-drug user over a 2-year period during adolescence. Arch Gen Psychiatry 67: 85–93. 20048226 10.1001/archgenpsychiatry.2009.173

[82] Griffin JP, Holliday RC, Frazier E, Braithwaite RL (2009) The BRAVE (Building Resiliency and Vocational Excellene) Program: Evaluation findings for a career-oriented substance abuse and violence prevention intervention. J Health Care Poor Underserved 20: 798–816. 19648706 10.1353/hpu.0.0174

