# Supplementary material for: Primary Prevention of Cannabis Use: A Systematic Review of Randomized Controlled Trials
Source: PLoS One. 2013 Jan 11;8(1):e53187. doi: 10.1371/journal.pone.0053187 (PMC3543459; doi:10.1371/journal.pone.0053187)
Supplement: Appendix S2 — Quality Criteria (adapted from EPOC Risk of Bias). (DOCX) [file pone.0053187.s002.docx]

Appendix S2

| Criterion | Description |
| --- | --- |
| Baseline Outcomes | Primary outcomes measured prior to intervention commencement and equivalence across intervention arms or otherwise adequately addressed |
| Baseline characteristics | Equivalence in participant characteristics across intervention groups or otherwise adequately addressed |
| Incomplete data (missing data & attrition) | Dropout or missing data minor or adequately addressed |
| Contamination | Control and intervention groups kept independent of each other |
| Selective reporting | All relevant outcome measures of the method section are reported in the results section |
| Blinding | Assessors were blind to intervention condition |
| Reliability of outcome measures | Outcome measures were adequate to assess the construct - at least 3 items or were they psychometrically validated |
| Adherence | Intervention implemented as intended by the project co-ordinators or adequately addressed |
| Exposure | Target group was exposed as specified to the intervention or adequately addressed |
