# Supplementary material for: Primary Prevention of Cannabis Use: A Systematic Review of Randomized Controlled Trials
Source: PLoS One. 2013 Jan 11;8(1):e53187. doi: 10.1371/journal.pone.0053187 (PMC3543459; doi:10.1371/journal.pone.0053187)
Supplement: Appendix S3 — List of excluded eligible studies (n = 21). (DOCX) [file pone.0053187.s003.docx]

Appendix S3

Bell RM, Ellickson PL, Harrison ER (1993) Do drug prevention effects persist into high school? How project ALERT did with ninth graders. Prev Med 22: 463-483.

Biglan A, Ary DV, Smolkowski K, Duncan T, Black C (2000) A randomised controlled trial of a community intervention to prevent adolescent tobacco use. Tob Control 9: 24-32.

Brown EC, Catalano RF, Fleming CB, Haggerty KP, Abbott RD (2005) Adolescent substance use outcomes in the Raising Healthy Children Project: A two-part latent growth curve analysis. J Consult Clin Psychol 73: 699 - 710.

Clayton RR, Cattarello AM, Johnstone BM (1996) The effectiveness of Drug Abuse Resistance Education (project DARE): 5-year follow-up results. Prev Med 25: 307-318.

Eddy JM, Reid JB, Stoolmiller M, Fetrow RA (2003) Outcomes during middle school for an elementary school-based preventive intervention for conduct problems: Follow-up results from a randomized trial. Behav Ther 34: 535-552.

Eisen M, Zellman GL, Massett HA, Murray DM (2002) Evaluating the Lions-Quest "Skills for Adolescence" drug education program: first-year behavior outcomes. Addict Behav 27: 619 - 632.

Eisen M, Zellman GL, Murray DM (2003) Evaluating the Lions-Quest "Skills for Adolescence" drug education program: second-year behavior outcomes. Addict Behav 28: 883 - 897.

Ellickson PL, Bell RM (1990) Drug prevention in junior high: a multi-site longitudinal test. Science 247: 1299 - 1305.

Ellickson PL, Bell RM, McGuigan K (1993) Preventing adolescent drug use: long-term results of a junior high program. Am J Public Health 83: 856-861.

Fearnow-Kenney MD, Wyrick DL, Jackson-Newso J, Wyrick CH, Hansen WB (2003) Initial indicators of effectiveness for a high school drug prevention program. Am J Health Educ 34: 66-71.

Furr-Holden CD, Ialongo NS, Anthony JC, Petras H, Kellam SG (2004) Developmentally inspired drug prevention: middle school outcomes in a school-based randomized prevention trial. Drug Alcohol Depend 73: 149-158.

Graham JW, Johnson CA, Hansen WB, Flay BR, Gee M (1990) Drug use prevention programs, gender, and ethnicity: evaluation of three seventh-grade Project SMART cohorts. Prev Med 19: 305-313.

Haggerty KP, Skinner M, Fleming CB, Gainey RR, Catalano RF (2008) Long-term effects of the Focus on Families project on substance use disorders among children of parents in methadone treatment. Addiction 103: 2008-2016.

Hansen WB, Graham JW (1991) Preventing alcohol, marijuana, and cigarette use among adolescents: peer pressure resistance training versus establishing conservative norms. Prev Med 20: 414-430.

Moore MJ, Werch CC (2009) Efficacy of a brief alcohol consumption reintervention for adolescents. Subst Use Misuse 44: 1009-1020.

Rohrbach LA, Gunning M, Sun P, Sussman S (2010) The Project Towards No Drug Abuse (TND) dissemination trial: implementation fidelity and immediate outcomes. Prev Sci 11: 77-88.

Schinke SP, Tepavac L, Cole KC (2000) Preventing substance use among Native American youth: three-year results. Addict Behav 25: 387-397.

Sloboda Z, Stephens RC, Stephens PC, Grey SF, Teasdale B, et al. (2009) The Adolescent Substance Abuse Prevention Study: A randomized field trial of a universal substance abuse prevention program. Drug Alcohol Depend 102: 1-10.

Smith EA, Swisher JD, Vicary JR, Bechtel LJ, Minner D, et al. (2004) Evaluation of Life Skills Training and Infused-Life Skills Training in a rural setting: Outcomes at two years. J Alcohol Drug Educ 48: 51-70.

Spoth R, Redmond C, Shin C, Azevedo K (2004) Brief family intervention effects on adolescent substance initiation: school-level growth curve analyses 6 years following baseline. J Consult Clin Psychol 72: 535-542.

St Pierre TL, Osgood DW, Mincemoyer CC, Kaltreider DL, Kauh TJ (2005) Results of an independent evaluation of Project ALERT delivered in schools by Cooperative Extension. Prev Sci 6: 305-317.
