# Supplementary material for: Primary Prevention of Cannabis Use: A Systematic Review of Randomized Controlled Trials
Source: PLoS One. 2013 Jan 11;8(1):e53187. doi: 10.1371/journal.pone.0053187 (PMC3543459; doi:10.1371/journal.pone.0053187)
Supplement: Appendix S1 — Search strategy for CINAHL (Ovid) 1987–2011. Limits included English language, peer reviewed and human. (DOCX) [file pone.0053187.s001.docx]

Appendix S1

1) cannabis (cannabis sativa)

2) *cannabis

3) *marijuana

4) *tetrahydrocannabinol

5) *THC

6) 1 or 2 or 3 or 4 or 5

7) *adolescent

8) *adolescence

9) *child

10) *youth

11) *student

12) adolescence

13) students

14) 7 or 8 or 9 or 10 or 11 or 12 or 13

15) *prevention

16) *early intervention

17) *program evaluation

18) *school based

19) Preventive health care

20) Preventive trials

21) Early intervention

22) Program evaluation

23) Schools

24) Elementary schools

25) Middle schools

26) Secondary schools

27) 15 or 16 or 17 or 18 or 19 or 20 or 21 or 22 or 23 or 24 or 25 or 26

28) 6 and 14 and 27
